# TAM receptor tyrosine kinases as potential mediators of the non-lytic spread of non-enveloped viruses

**DOI:** 10.1042/BST20250119

**Published:** 2026-05-08

**Authors:** Steven J. Moran, Chrystal A. Starbird

**Affiliations:** 1 Department of Biochemistry and Biophysics, University of North Carolina School of Medicine, Chapel Hill, NC 27599, U.S.A.; 2 Lineberger Comprehensive Cancer Center, University of North Carolina School of Medicine, Chapel Hill, NC 27599, U.S.A.

**Keywords:** non-lytic spread, receptor tyrosine kinases, signalling, TAM Receptors, viral spread

## Abstract

Tyro3, Axl, and Mer are receptor tyrosine kinases that comprise the TAM receptor family. These receptors, originally identified as orphan receptors, do not bind growth factors but rather ligands that can facilitate processes such as phagocytosis and dampening the innate inflammatory immune response. Enveloped viruses can hijack TAM receptors for viral entry through the fairly well-established mechanism of apoptotic mimicry. This mechanism involves ‘tricking’ the targeted host cell into endocytosing the virus through binding to exposed phosphatidylserine in the viral lipid bilayer envelope. While enveloped viruses utilize apoptotic mimicry for entry, it remains unclear how non-enveloped viruses enter the host cell through phosphatidylserine receptors such as TAM receptors. There is evidence that non-enveloped viruses can usurp host cell signaling pathways to cloak their particles in membranes, creating ‘quasi-enveloped’ viruses that enter the host through a sort of ‘faux apoptotic mimicry.’ These quasi-enveloped viruses can spread through non-lytic mechanisms to evade the host immune response and deliver virus particles to the targeted host cell. With the present review, we evaluate increasing evidence that TAM receptors may play a role in this process through their ability to indiscriminately bind phosphatidylserine in membranes, leading to the internalization of non-enveloped viruses packaged within phosphatidylserine-enriched extracellular vesicles.

## Introduction

Organismal homeostasis, development, and disease progression are examples of key processes regulated through cell-to-cell communication [1]. This communication is typically initiated through ligands binding to membrane-bound receptors and propagating a signaling cascade within the cell [2]. These signaling cascades mediate cellular responses that can influence cell shape and function [3]. Notable membrane-bound receptors that are critical in human health and function are receptor tyrosine kinases (RTKs). RTKs are a diverse family of plasma membrane receptors capable of binding ligands such as growth factors, hormones, and cytokines to mediate cellular and metabolic signaling pathways [4]. Initially identified as receptors for insulin and epidermal growth factor (EGF), RTKs have now been established as model receptors for understanding cell signaling pathways [4].

Within the RTK family are a subfamily of receptors collectively known as TAM receptors. The name of this family is derived from the first letter of each family member: Tyro3, Axl, and Mer [5]. In the early 1990s, TAM receptors were first grouped into a distinct RTK family named the Tyro3/7/12 cluster based on PCR cloning of their kinase domains. The isolation of the full-length cDNAs for Axl, Mer, and Tyro3 then allowed for them to be categorized into a structurally distinctive family believed to be ‘orphan’ RTKs [5]. However, it was soon discovered that TAM receptors do interact with ligands that bind and activate these receptors: growth arrest-specific gene 6 (Gas6) and protein S (Pros1) [5]. Gas6 shares about 40% amino acid sequence identity with Pros1, an abundant serum protein that plays a role in regulating blood coagulation [6,7]. Additional potential ligands have been discovered that potentially interact with TAM receptors. TUBBY, TUBBY-like protein (TULP-1), and galectin-3 (Gal3) have been shown to activate Mer and induce phagocytosis of the retinal pigment epithelium [8]. TULP-1 has also been shown to interact with Tyro3 and Axl, but the biochemical implications of their interactions remain unclear [6,8]. Feimin, a secreted skeletal muscle protein that regulates glucose homeostasis, has recently been discovered as a ligand for Mer [9]. When bound to Mer, feimin promotes glucose uptake and inhibits glucose production through Akt activation [9]. Future studies are needed to fully understand the role of these additional ligands in the context of TAM receptor binding and activity.

The lack of an essential role in embryonic development further distinguishes TAM receptors from other RTKs [5]. Single murine loss-of-function mutations of TAM receptors have demonstrated that mice remain viable and fertile 2–3 weeks after birth [5,6]. In contrast, double- or triple-TAM knockout (KO) animals normally develop but show defects in efferocytosis, the process of apoptotic cell clearance through phagocytosis; accumulate apoptotic cells; have lymphocytes activated in multiple tissues; elevated pro-inflammatory cytokine levels; and auto-antibody production [6,10]. These abnormalities have led to the development of human-like autoimmune conditions such as systemic lupus erythematous, psoriasis, rheumatoid arthritis, nephritis, and multiple sclerosis, specifically in TAM KO mice with triple mutations [11,12]. Blindness and male infertility were also observed in mice due to impaired photoreceptor segment phagocytic clearance in the retinal pigment epithelium, and impaired Sertoli cell phagocytic activity disrupting spermatogenesis, respectively [10,13,14].

TAM receptors are expressed on the cell surface of sentinel immune cells, vascular endothelial cells, neuronal cells and glia, and professional phagocytic immune, nervous, and reproductive cells [15]. They primarily function in mediating ‘homeostatic’ or silent phagocytic clearance of apoptotic cells resulting from normal cell turnover and in attenuating the innate inflammatory immune response in phagocytic and sentinel immune cells [15,16]. Aberrant expression of these receptors has been implicated in the progression of various pathologies such as cancer and autoimmune disease [10,17–19]. It has also been shown that aberrant TAM receptor expression can increase susceptibility to viral infection. Induced expression of TAM receptors and ligands can enhance the entry of pathogenic viruses with a host cell-derived lipid bilayer envelope [15,20–23]. While this has increasingly become more established for this type of virus, it remains unclear whether viruses without a host cell-derived lipid bilayer envelope (i.e., non-enveloped viruses) can also similarly utilize TAM receptors and ligands for entry. The present review will focus on the current literature on TAM receptors and their roles in enveloped and non-enveloped virus infection and spread.

## Structure and function of TAM receptors and ligands

Post-translational modifications such as glycosylation, phosphorylation, and ubiquitination make full-length, mature human Tyro3 and Axl have molecular weights between 100 and 140 kDa, while Mer has a molecular weight between 165 and 205 kDa [24]. Like other RTKs, TAM receptors consist of an ectodomain, transmembrane domain, and a conserved intracellular kinase domain [10]. Located within the kinase domain of TAM receptors is a conserved sequence, KW(I/L)A(I/L)ES, that further distinguishes TAM receptors from other RTKs [6,17,18]. The amino-terminal region of the ectodomain contains tandem immunoglobulin (Ig)-like repeats followed by tandem fibronectin type III (FNIII) repeats that facilitate ligand binding [5]. This is then followed by a single-pass transmembrane domain that passes through the plasma membrane and connects with the catalytically competent intracellular tyrosine kinase domain [5]. The kinase domain has activation loops containing three tyrosine residues that act as sites for receptor phosphorylation [10]. Ligand binding to the TAM receptor is thought to trigger receptor and ligand dimerization or clustering, and subsequent cross-phosphorylation of the kinase domains. Secondary trans-phosphorylation of the kinase domains, juxta-membrane, and carboxy-terminal regions then allows for the recruitment of intracellular substrates for downstream signaling pathways [4,18].

Gas6 and Pros1 ligands are vitamin K-dependent proteins that have predicted molecular weights of 75 and 73 kDa, respectively [25,26]. Both Gas6 and Pros1 have an amino-terminal gamma-carboxyglutamic acid (Gla) domain that is followed by a thrombin-sensitive loop region and four subsequent EGF-like domains [25]. While they share high structural homology, Gas6 is distinguished from Pros1 through possessing a thrombin-sensitive disulfide-bridged thumb loop that is not susceptible to serine protease cleavage [25,27]. The carboxy-terminal end of both ligands consists of two laminin G repeats that comprise the sex hormone-binding globulin (SHBG) domain [13]. This particular region is integral for TAM receptor binding and phosphorylation [5]. Gas6 has a higher binding affinity to Axl and Tyro3 relative to Mer within a single- to double-digit nanomolar range, while Pros1 has been demonstrated to preferentially bind to Tyro3 over Mer over a wider nanomolar range [15,28]. Several studies have provided compelling evidence that Pros1 does not bind Axl, but some studies have suggested that Pros1 still has an impact on Axl expression [15,25,29,30].

The plasma membrane of eukaryotic cells has an asymmetrical distribution of phospholipids [31]. The inner leaflet of the plasma membrane primarily contains phosphatidylserine (PtdSer) and phosphatidylethanolamine (PtdEtn), while phosphatidylcholine (PtdCho) and sphingomyelin (SM) are in the outer leaflet. The distribution of these phospholipids is dependent on the activity of phospholipid translocases such as flippases, floppases, and scramblases [32]. When a cell is undergoing cell death (i.e., apoptosis), the redistribution of PtdSer from the inner leaflet of the plasma membrane to the outer leaflet primes the cell for phagocytosis [33].

**Figure 1:**
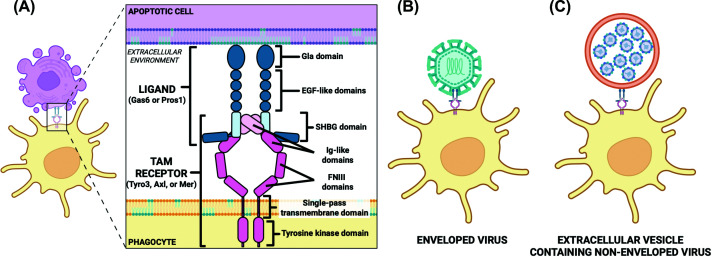
TAM receptor/ligand complex binding to membrane phosphatidylserine (**A**) TAM (Tyro3, Axl, and Mer) receptors (shades of pink) expressed on the surface of phagocytes are activated when bound by ligands (Gas6 or Pros1, shades of blue). Upon ligand binding and activation, a bridging complex is formed that allows for engagement with phosphatidylserine (PtdSer) in the membrane of the target apoptotic cell. Phagocytosis is induced, and the apoptotic cell is cleared from the extracellular environment. This routine engulfment process does not elicit an immune response because of TAM receptor signaling dampening innate inflammatory immune responses. (**B**) Enveloped viruses like Zika virus can ‘trick’ a phagocyte expressing an activated TAM receptor/ligand complex into phagocytosing them by having host cell-derived PtdSer displayed on the outer leaflet of their viral envelope, mimicking an apoptotic cell. This mechanism is called apoptotic mimicry. (**C**) Non-enveloped viruses like poliovirus can package their particles into a PtdSer-enriched extracellular vesicle and enter a naïve cell to establish infection. It is possible that non-enveloped viruses use activated TAM receptor/ligand complexes to enter through a ‘faux apoptotic mimicry’ mechanism. Gas6: growth arrest-specific 6; Pros1: Protein S; EGF: epidermal growth factor; SHBG: sex hormone-binding globulin; Ig: immunoglobulin; and FNIII: fibronectin type III.

During phagocytic clearance of apoptotic cells, TAM receptors and their ligands form a ‘phagocytic synapse’ (Figure 1A). This synapse is formed by an interaction between a TAM receptor/ligand complex on the surface of a phagocyte bound to externalized PtdSer on an apoptotic cell [13]. The carboxy-terminal SHBG domain of Gas6/Pros1 binds to Ig-like repeats in the TAM receptor ectodomain, which is thought to induce receptor oligomerization and subsequent cross-phosphorylation of the intracellular tyrosine residues [13]. This signaling event primes the phagocyte to initiate phagocytosis. Vitamin K-dependent gamma-carboxylation of the amino-terminal Gla domains of Gas6/Pros1 is a requirement for Ca^2+^ binding, a necessity for complete activation of TAM receptors. Non-carboxylated or truncated Gla domains can still bind ligands but cannot potentiate TAM receptor activation [6,15]. The fully activated TAM receptor with bound ligand can then bind to the ‘eat-me’ signal PtdSer on the targeted apoptotic cell, triggering signaling cascades that remodel the actin cytoskeleton and allow for targeted internalization [13,34].

TAM receptor signaling is also important in regulating innate inflammatory immune responses [19]. Toll-like receptor and type I interferon (IFN) signaling are known to activate TAM receptor signaling in phagocytes such as macrophages and dendritic cells. When this signaling is dysregulated, it can lead to chronic inflammation and potentially autoimmune disease [35]. TAM receptor signaling prevents this dysregulation through immunosuppression. It has been shown that the co-expression of Axl and IFN receptors in macrophages and dendritic cells inhibits innate inflammatory immune responses [36]. This mechanism was determined in dendritic cells in which Axl heterodimerization with the type I IFN receptor (IFNAR) induced expression of genes encoding suppressor of cytokine signaling 1 (SOCS1) and 3 (SOCS3), resulting in the inhibition of pro-inflammatory cytokine release and promotion of immunosuppression [36,37].

## Enveloped virus spread through apoptotic mimicry

It has been established with enveloped viruses that TAM receptors play a role in viral infection [15,23]. Enveloped viruses contain a lipid bilayer envelope derived from the host cell that is typically decorated in viral glycoproteins. These viral glycoproteins permit attachment and facilitate entry through endocytosis or fusion of the viral envelope with the host plasma membrane [38]. Enveloped viruses such as Ebola virus and Marburg virus (*Filoviridae*) [20], Zika virus and dengue virus (*Flaviviridae*) [22,39], and Lassa virus (*Arenaviridae*) [40] have been shown to utilize TAM receptors to facilitate viral infection.

Enveloped viruses primarily use TAM receptors to facilitate entry into host cells through apoptotic mimicry (Figure 1B). This mechanism was first proposed in hepatitis B virus-infected hepatocytes that generated hepatitis B surface antigen, non-infectious subviral particles rich in the lipid PtdSer that aid in immune evasion [41]. The display of PtdSer marks a dying cell for phagocytic clearance through TAM receptor-mediated signaling [13,34]. Enveloped viruses can mimic the apoptotic state of a dying cell by incorporating PtdSer into the outer leaflet of their host cell-derived lipid bilayer envelope. This allows the virus to ‘trick’ phagocytes into engulfing the virus [41–44].

The utilization of apoptotic mimicry is dependent on the distribution of PtdSer and/or PtdEtn in the outer leaflet of the viral envelope [41]. Enveloped viruses have developed mechanisms that induce the reorganization of PtdSer and PtdEtn from the inner leaflet to the outer leaflet to aid in the production of progeny virions that can utilize apoptotic mimicry [41]. While many viral infections cause cell death and increased PtdSer in the inner leaflet of the plasma membrane, some infections can activate scramblases to enhance virion infectivity. Ebola virus infection *in cellulo* showed an increase in PtdSer externalization through activation of the scramblase transmembrane protein 16F (TMEM16F) [45]. This is supported by other evidence showing that the lack of apoptosis-induced scramblase XK-related protein 8 (XKR8) led to the synthesis of less infectious Ebola virus particles that contained lower levels of PtdSer in their envelopes [46,47].

During viral entry through apoptotic mimicry, TAM receptors do not directly function as virus receptors, but rather Gas6 and Pros1 act as bridging molecules between the viral envelope and the target host cell expressing the TAM receptor/ligand complex [5]. The enveloped virus still uses its primary receptor for entry and subsequent infection. The indirect utilization of TAM receptors for entry and the dampening of innate inflammatory immune responses upon TAM receptor activation aids viral replication and immune evasion [48]. All three TAM receptors have been shown to act as entry factors for Ebola and Marburg filoviruses [20,49]. Lymphoid cells resistant to filovirus infection became susceptible to infection with pseudotyped viruses containing filovirus envelope glycoproteins when ectopic TAM receptor expression was induced. This enhanced infectivity was abrogated when treated with antibodies targeting full-length and soluble TAM receptors and Gas6 [20]. Functional studies with Axl mutants also showed that filovirus entry is dependent on the physiological functions of Axl when mediating infection through this specific TAM receptor [49]. The role of Axl in enveloped virus infection was further investigated with pseudotyped lentiviral vectors expressing Sindbis virus (*Togaviridae*) envelope glycoproteins. When assessing the residual infectivity of the pseudotyped virus, it was determined that Gas6 and Pros1 enhanced pseudotyped virus infectivity by acting as bridging molecules between PtdSer on the viral envelope and Axl on the targeted host cell [21]. The viral infectivity of dengue virus has also been shown to be enhanced by TAM receptor expression, specifically Tyro3 and Axl expressed in HEK293T cells [22]. It was suggested that dengue virus particle recognition by Tyro3 or Axl is mediated by Gas6 binding viral envelope PtdSer and either receptor [22]. The involvement of Gas6 specifically acting as a bridging molecule between enveloped virus particles and TAM receptors has been further investigated with dengue virus, West Nile virus, and Zika virus. The higher PtdSer content in the Zika virus envelope allowed it to more favorably bind Gas6 relative to dengue virus and West Nile virus with lower PtdSer content in their envelopes [50].

While apoptotic mimicry has been mostly established with enveloped viruses containing a viral RNA genome, this mechanism has also been utilized by other enveloped viruses such as vaccinia virus. Vaccinia virus is an enveloped DNA virus belonging to *Poxviridae*, the family that houses the poxvirus known to have caused smallpox [51]. During replication, vaccinia virus forms infectious intracellular mature virus (MV) particles that acquire their membrane from inside the target host cell and are released through cell lysis [21]. These particles are similar in size to apoptotic cells and contain PtdSer in their membranes [52]. It has been suggested that the entry of these particles through macropinocytosis and their composition mimicking apoptotic cells allows vaccinia virus to use apoptotic mimicry for potential immune evasion [52]. Vaccinia extracellular enveloped virus (EEV) particles, infectious MV particles comprised of an additional membrane derived from the *trans*-Golgi or early endosome membranes, have been shown to use human Gas6 (hGas6) for host cell entry [21]. This was supported through additional experiments showing that incubating human microvascular endothelial cells with anti-Axl antibody or annexin V (ANX5) blocks hGas6-mediated enhancement of EEV infection [21].

Of note, other PtdSer receptors have been identified that appear to impact viral entry. One example is the T-cell immunoglobulin mucin (TIM) family, which consists of proteins involved in the development of autoimmunity and allergic diseases [53]. TIM receptors also function as PtdSer receptors, and a direct relationship between TIM and TAM receptors has been proposed in normal cells, whereby TIM receptors appear to enhance TAM-mediated phagocytosis [54]. Similarly, stable expression of TIM1 and TIM4 in HEK293T cells has been shown to enhance dengue virus infection relative to the minimal infection observed in wild-type cells [22]. The effects of enhanced infection were attenuated through the use of anti-TIM1 and anti-Axl antibodies, suggesting that dengue virus requires both TIM and TAM receptors to recognize PtdSer for infection [22]. TIM1 has also been implicated in Ebola virus infection. Ectopic expression of TIM1 has been shown to enhance Ebola virus infection, while treatment with the ARD5 monoclonal antibody that blocks TIM1 binding to PtdSer blocks virus binding and infection [55].

CD300a, a phospholipid receptor of the CD300 family of activating/inhibitory receptors, has been shown to directly bind dengue virus particles and enhance viral entry through clathrin-mediated endocytosis [56,57]. This was observed in HEK293T cells, while antibodies against CD300a have shown to partially inhibit dengue virus entry in macrophages [57]. Milk fat globule-EGF factor 8 protein (MFG-E8), also known as lactadherin, is a PtdSer-binding protein that can initiate phagocytic clearance of apoptotic cells [41,58]. Although it was shown to bind rotavirus (*Sedoreoviridae)* and prevent infection, MFG-E8 was not categorized as an enhancement factor for enveloped virus entry until it was discovered to enhance pseudo-particle entry [59]. It remains unclear whether TAM receptors and ligands played a role in these cases of viral entry mediated by CD300a and MFG-E8.

While PtdSer receptors can aid in viral entry, they can also limit virus spread. Cells that express PtdSer receptors on their surface can bind newly budded virions and prevent their release, similar to the function of tetherin ‘tethering’ budded human immunodeficiency virus type 1 (HIV-1) particles to cells [41,60]. It has been demonstrated through transmission electron microscopy that HEK293T cells co-transfected with proviral HIV-1 DNA and TIM1 plasmid had more mature HIV-1 particles accumulate on the cell surface than cells not expressing TIM1 [61]. These results were further supported with TIM1 mutants deficient in PtdSer binding having a significantly decreased ability to inhibit HIV-1 particle release relative to cells expressing wild-type TIM1 [61]. The virally encoded negative factor (Nef) protein can counteract this inhibitory effect by inducing the internalization of TIM receptors like TIM1 [62]. This phenomenon was also observed with Chikungunya virus, in which virus particle release significantly decreased when TIM1 was expressed on the host cell surface [63]. This effect could only be rescued through direct TIM1 KO or altering lipid composition [63]. These cases provide evidence that PtdSer-binding receptors such as TIMs and TAMs may have an alternative inhibitory effect on viral entry depending on the virus. Immune responses elicited upon viral infection vary among viruses, which could also dictate whether a PtdSer-binding receptor will promote or inhibit viral entry.

## Non-enveloped virus spread through vesicle-mediated en bloc transmission

There is an emerging interest in understanding the role of PtdSer receptors like TAM receptors in non-enveloped virus entry. While the role of PtdSer receptors in enveloped virus entry is more established, there is mounting evidence that non-enveloped viruses have adapted to using PtdSer receptors to facilitate entry into target host cells (Figure 1C). Non-enveloped viruses do not typically derive a lipid bilayer envelope upon exit from the targeted host cell. However, it has been shown that some non-enveloped viruses hijack cellular exosome machinery to initiate infection among cells [64]. These quasi-enveloped viruses contain membranes with PtdSer that can facilitate entry through PtdSer receptors [41]. This mode of viral transmission has been termed ‘vesicle-mediated en bloc transmission’ [65–67]. Vesicle-mediated en bloc transmission as a mode for viral transmission allows for multiple viral genomes to enter and initiate a productive infection in the targeted host cell [68]. It also allows viruses to evade the host immune response and enable non-lytic spread to enhance virulence [68]. Although most viruses execute a lytic replication program that allows for virus budding and subsequent cell lysis, some undergo a non-lytic program that allows for viral spread to occur without cell lysis [69].

Many non-enveloped viruses have been shown to undergo non-lytic spread using extracellular vesicles as an additional mechanism of entry that enhances virulence. JC polyomavirus (*Polyomaviridae*), a DNA virus associated with progressive multifocal leukoencephalopathy, creates extracellular vesicles containing infectious particles that protect the virus from antibody neutralization and allow for transmission independent of viral entry receptors lactoseries tetrasaccharide C and 5-hydroxytryptamine [68,70–72]. Gastrointestinal RNA viruses rotavirus and norovirus (*Caliciviridae*) are known to be packaged in extracellular vesicles for vesicle-mediated en bloc transmission. Extracellular vesicles containing rotavirus particles are derived from the plasma membrane, while vesicles containing norovirus are derived from PtdSer-containing multivesicular bodies that are released through the exosomal pathway [67]. Both of these quasi-enveloped viruses are shed in feces and remain infectious when transmitted through the fecal-oral route [67]. Hepatitis E virus (*Hepeviridae*) circulates in human blood in a quasi-enveloped form while its non-enveloped form is shed in feces [73]. Quasi-enveloped hepatitis E virus hijacks the endocytic pathway for entry through clathrin-mediated endocytosis and trafficking to an acidic compartment within the lysosome to degrade the quasi-enveloped particle prior to penetration [73]. While still infectious, quasi-enveloped hepatitis E virus requires a longer inoculation period than its non-enveloped form due to the lack of viral attachment proteins [73].

Non-enveloped viruses such as hepatitis A virus and poliovirus (*Picornaviridae*) have also been shown to utilize this mechanism of spread. Hepatitis A virus can cloak itself in cellular membranes that closely resemble exosomes, and the replication of these quasi-enveloped hepatitis A virus particles was abrogated through treatment with anti-capsid antibodies [74]. The hijacking of host cellular membranes by hepatitis A virus for protection and viral spread essentially removes the distinction between enveloped and non-enveloped viruses [74]. Poliovirus, the enteric virus that causes poliomyelitis, is known to remodel intracellular membranes and lipid pools during infection to create replication organelles rich in phosphatidylinositol-4-phosphate (PtdIns4P) and cholesterol [75]. It can replicate and assemble virions in these organelles that are then packaged into ER-derived autophagosomes positive for the autophagy marker microtubule-associated protein 1A/1B light chain 3B (LC3) and enriched in PtdSer [66,69]. These atypical autophagosomes package approximately 20–30 immature poliovirus particles that gradually mature through lumen acidification as they travel toward the plasma membrane [66,76]. Unlike typical autophagosomes that fuse with lysosomes, these autophagosomes fuse with the plasma membrane and allow for the release of poliovirus particles in LC3-positive, PtdSer-enriched extracellular vesicles [66,68].

The degree to which polioviruses and other enteroviruses utilize non-lytic spread in hosts is less clear, but it is clear that the host autophagy pathway appears to be critical for this mechanism of viral spread [69,77,78]. Although poliovirus is thought to be predominantly a lytic virus, it has been shown through treatment with autophagy-stimulating drugs loperamide and nicardipine that poliovirus non-lytic spread is enhanced *in cellulo* and is more pathogenic *in vivo*. This enhancement was attenuated *in cellulo* when LC3 expression was silenced through RNAi treatment, providing further evidence of poliovirus non-lytic spread being dependent on autophagy [69]. It has recently been demonstrated that poliovirus utilizes the virally encoded nonstructural protein 3CD to mediate non-lytic spread through mediating particle movement and packaging into autophagosomes and regulating the formation of autophagosomes. Poliovirus 3CD was also shown to mediate the movement of poliovirus-packaged autophagosomes to the plasma membrane for eventual release through exocytosis [79]. This was determined through the mutagenesis of the LC3-interaction regions of 3CD, causing a severe defect in non-lytic spread by preventing particle incorporation into LC3-positive autophagosomes and particle trafficking to the plasma membrane [79].

## The role of TAM receptors in the non-lytic spread of non-enveloped viruses

While it has not yet been determined, there is increasing evidence that PtdSer receptors such as TAM receptors may play a role in the non-lytic spread of non-enveloped viruses. With enveloped viruses such as Zika virus, extracellular vesicles containing PtdSer have been shown to co-localize with Axl and competitively interfere with viral entry through apoptotic mimicry [44]. Axl bound by these extracellular vesicles was also shown to be internalized along with the vesicles [44]. It is possible that non-enveloped virus particles packaged into PtdSer-containing extracellular vesicles could co-localize and bind to an active TAM receptor/ligand complex, inducing internalization of the vesicle–receptor complex for viral entry.

There are two non-enveloped viruses known to interact with PtdSer receptors: hepatitis A virus and murine norovirus. It was thought that hepatitis A virus entry was co-facilitated by TIM1 due to anti-TIM1 monoclonal antibodies blocking viral entry [80]. However, it has been demonstrated that TIM1 is not essential for the entry of quasi-enveloped or naked hepatitis A virus particles [81]. Rather, TIM1 could possibly aid in the internalization of quasi-enveloped hepatitis A virus particles and traffic the naked form of those particles to different compartments within the endocytic pathway [81]. The potential role of TIM1 in hepatitis A virus particle internalization is supported by evidence in which TIM1 functioning with the cholesterol transporter Niemann-Pick C1 (NPC1) helped to deliver exosomes packaged with hepatitis A virus particles to the host cell through clathrin-mediated endocytosis [64]. Murine norovirus infection *in cellulo* and *in vivo* has been shown to be mediated through binding CD300lf, a member of the CD300 family [82]. Wild-type BV2 microglial cells infected with different murine norovirus strains developed cytopathic effects relative to CD300lf-deficient cells. HeLa cells, normally unsusceptible to murine norovirus infection, were also susceptible to infection when CD300lf was overexpressed [82]. Mice expressing CD300lf were also susceptible to infection with different murine norovirus strains, as demonstrated through the detection of viral genomes shed in the feces of some of the mice. Interestingly, human CD300lf was found to be nonessential for human norovirus infection, nor did it prevent the binding of human norovirus-like particles to host cell glycans [82]. Other non-enveloped viruses could possibly indirectly or directly use PtdSer receptors and additional non-native receptors for entry. Whether those viruses will undergo non-lytic spread through extracellular vesicles remains unclear.

The coordinative activity of TAM receptors and native virus entry receptors should be considered during non-enveloped virus entry. Enveloped viruses like severe acute respiratory syndrome coronavirus 2 (SARS-CoV-2) have been shown to utilize PtdSer receptors of the TIM (TIM1 and TIM4) and TAM (Axl) families for enhanced entry in cells expressing low levels of the host entry receptor angiotensin-converting enzyme 2 (ACE2) [83]. When investigating the role of Axl in SARS-Co-V-2 entry, it was shown that the SARS-Co-V-2 viral attachment protein did not directly interact with Axl. Rather, Axl interacted with the PtdSer on the envelope of SARS-Co-V-2 particles, reminiscent of apoptotic mimicry [83]. The use of PtdSer receptors when the native virus entry receptor is lowly expressed suggests that unfavorable conditions for the virus could induce changes in viral entry strategy. The initiation of infection could start with attachment and entry through PtdSer receptors like TAM receptors, and either continue along with the native virus entry receptor when conditions are favorable or continue solely with the native virus entry receptor once a productive infection has been established. This could be extended to non-enveloped viruses, which have created unique ways of facilitating infection and spread through non-lytic mechanisms such as vesicle-mediated en bloc transmission. Other factors that could influence non-enveloped viruses to utilize TAM receptors for non-lytic spread are the activity of other PtdSer receptors like TIM receptors, the actin cytoskeleton, the abundance of activating ligand, and PtdSer content in the vesicles encapsulating non-enveloped virus particles.

PtdSer content has specifically been shown to be a determining factor in the viral infection and spread of some enveloped and non-enveloped viruses. The increased abundance of PtdSer in the envelope of Zika virus allowed it to readily bind the TAM-activating ligand Gas6 relative to dengue virus and West Nile virus [50]. It is possible that extracellular vesicles containing PtdSer and non-enveloped virus could associate with TAM receptors and initiate entry through a ‘faux-apoptotic mimicry’ mechanism, leading to non-lytic spread from cell to cell. Non-lytic spread involving PtdSer and TAM receptors could allow non-enveloped viruses to evade the host immune response while supplying the targeted host cell with enough virus for a productive infection. This mechanism could explain the heightened virulence of some non-enveloped viruses.

## Conclusions

**Figure 2:**
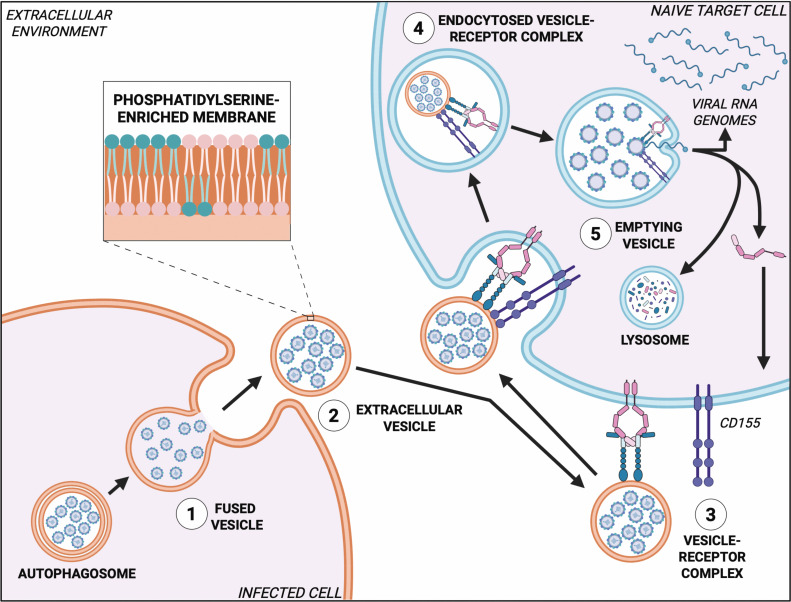
Model of ‘faux apoptotic mimicry’ as a mechanism for the non-lytic spread of poliovirus through TAM receptors (**1**) An autophagosome containing poliovirus particles fuses with the infected cell plasma membrane; (**2**) a newly budded extracellular vesicle packaged with poliovirus particles and enriched in phosphatidylserine (PtdSer) is released from the infected cell; (**3**) the vesicle binds to an activated TAM receptor/ligand complex expressed on a naïve target cell; (**4**) the vesicle–receptor complex is endocytosed into the naïve target cell with aid from the natively expressed poliovirus receptor CD155; and (**5**) poliovirus genome penetration occurs in the cytoplasm possibly mediated by an activated TAM receptor/ligand complex and CD155. Monomeric TAM receptor is recycled to the plasma membrane or degraded along with ligand and CD155 in the lysosome.

TAM receptors are a family of RTKs that are important for cellular homeostasis. Their activation and function are dependent upon binding the ligands Gas6 or Pros1. When activated upon ligand binding, the resulting TAM receptor/ligand complex can then bind to PtdSer and facilitate processes such as the phagocytic clearance of apoptotic cells and dampening the innate inflammatory immune response. The ubiquitous expression and regulatory functions of TAM receptors make them attractive targets for viral infection. While it has been established that enveloped viruses hijack TAM receptors for viral entry through apoptotic mimicry, it is still unclear how these receptors may aid in non-enveloped virus infection (Figure 2). Figure 2 presents a model of this process, but many of the details of vesicle entry, uncoating, and the release of viral genomes remain uncertain and require further investigation. Furthermore, it remains of interest how non-enveloped viruses may use TAM receptors for non-lytic spread. Future studies on the role of TAM receptors in non-enveloped virus infection and non-lytic spread could provide novel insights into alternative TAM receptor function and lead to the development of targeted antiviral therapies.

## Perspectives

- TAM receptors mediate the phagocytosis of apoptotic cell debris and the downregulation of innate inflammatory immune responses upon binding Gas6 or Protein S activating ligands.
- Enveloped viruses can use apoptotic mimicry to facilitate viral entry and immune silencing through TAM receptors, while non-enveloped viruses can enter as ‘quasi-enveloped’ viruses or be packaged into extracellular vesicles enriched in PtdSer for entry through ‘faux apoptotic mimicry.’
- Non-lytic spread of non-enveloped viruses through TAM receptors may be dependent on other components associated with phagocytosis, the availability of TAM ligands, and PtdSer content in extracellular vesicles containing non-enveloped virus particles.

## Open Access

Open access for this article is provided via our Subscribe to Open programme, made possible through the support of our subscribing institutions. Learn more here: https://portlandpress.com/pages/open_access_options_and_prices#conditional.

## Abbreviations

ACE2: angiotensin-converting enzyme 2
ANX5: annexin V
EEV: extracellular enveloped virus
EGF: epidermal growth factor
FNIII: fibronectin type III
Gal3: galectin-3
Gas6: growth arrest-specific gene 6
Gla: gamma-carboxyglutamic acid
hGas6: human Gas6
HIV-1: human immunodeficiency virus type 1
IFN: interferon
IFNAR: interferon receptor
Ig: immunoglobulin
KO: knockout
LC3: microtubule-associated protein 1A/1B-light chain 3
MFG-E8: milk fat globule-EGF factor 8 protein
MV: mature virus
Nef: negative factor
NPC1: Niemann-Pick C1
Pros1: protein S
PtdCho: phosphatidylcholine
PtdEtn: phosphatidylethanolamine
PtdIns4P: phosphatidylinositol-4-phosphate
PtdSer: phosphatidylserine
RTKs: receptor tyrosine kinases
SARS-CoV-2: severe acute respiratory syndrome coronavirus 2
SHBG: sex hormone-binding globulin
SM: sphingomyelin
SOCS1: suppressor of cytokine signaling 1
SOCS3: suppressor of cytokine signaling 3
TAM: Tyro3, Axl, and Mer
TIM: T-cell immunoglobulin mucin
TMEM16F: transmembrane protein 16F
TULP-1: TUBBY-like protein
XKR8: XK-related protein 8
